# Understanding continued use of smart learning platforms: psychological wellbeing in an extended TAM-ISCM model

**DOI:** 10.3389/fpsyg.2025.1521174

**Published:** 2025-05-14

**Authors:** Jinlei Li, Meilin Jin, Xiaowei Chen

**Affiliations:** ^1^Academic Affairs Office, Zhejiang Institute of Communications, Zhejiang, China; ^2^School of Digital Economics, Wenzhou Vocational College of Science and Technology, Zhejiang, China; ^3^College of Arts, Zhejiang Shuren University, Zhejiang, China; ^4^College of Creative Arts, Universiti Teknologi MARA, Shah Alam, Malaysia

**Keywords:** technology acceptance model, continuous use intension, psychological wellbeing, user engagement, perceived enjoyment, perceived trust

## Abstract

**Introduction:**

In response to the growing adoption of digital education technologies, this study explores the factors influencing the continuous use of innovative learning platforms among students in higher vocational education. Recognizing that technological performance and psychological experiences shape user engagement, this research extends the Technology Acceptance Model (TAM) by integrating constructs such as perceived trust, enjoyment, ideological alignment, and psychological states—including satisfaction, wellbeing, and anxiety.

**Methods:**

Data were collected from 782 higher vocational college students in China using an online questionnaire. Participants represented diverse academic fields (e.g., liberal arts, sciences, engineering, arts) and were balanced in terms of gender (44.28% male and 55.72% female). Structural Equation Modeling (SEM) was applied to assess direct and indirect relationships among key variables, including perceived usefulness, perceived ease of use, system quality, expectation confirmation, enjoyment, and psychological indicators such as anxiety and subjective wellbeing. The mediating role of psychological wellbeing was also tested to evaluate its influence on continued platform usage.

**Results:**

Perceived enjoyment emerged as the strongest predictor of continuance intention (*β* = 0.52, *p* < 0.001), underscoring the central role of affective engagement in promoting sustained platform use. Perceived usefulness (*β* = 0.38, *p* < 0.01) and expectation confirmation (*β* = 0.31, *p* < 0.01) also exerted significant positive effects, supporting the cognitive appraisal mechanisms outlined in TAM and Expectation-Confirmation Theory. Interestingly, perceived trust negatively affected satisfaction (*β* = −0.13, *p* < 0.05), possibly due to a mismatch between institutional trust and user expectations, which may lead to psychological strain. System quality had no significant impact on satisfaction (*β* = 0.05, *p* > 0.05), suggesting that users view platform reliability as a baseline requirement rather than a satisfaction driver. Moreover, psychological wellbeing—defined by higher satisfaction and lower anxiety—is mediated between platform experience and continued use.

**Discussion:**

These findings highlight the need for innovative learning platforms to address technological expectations and psychological resilience. While usability and usefulness remain essential, designers must foster emotional engagement and manage trust-based expectations. Platforms that overlook psychological dimensions risk diminishing user satisfaction and long-term retention.

**Conclusion:**

Sustaining engagement with innovative learning platforms in higher vocational education requires a holistic approach that balances functional usability with mental wellbeing. This study offers important theoretical and practical insights for educators, developers, and policymakers aiming to create emotionally supportive and pedagogically effective digital learning environments.

## 1 Introduction

In recent years, research on digital learning platforms has predominantly focused on factors influencing initial technology adoption. Although the Technology Acceptance Model (TAM) has been widely employed as a theoretical foundation in these studies (Davis, 1989), it was initially conceptualized to explain short-term acceptance rather than long-term usage behavior. Consequently, TAM alone may not sufficiently account for the complexities of sustained engagement, particularly in dynamic and psychologically demanding educational contexts where ongoing platform use is crucial for learning continuity and academic success (Venkatesh and Davis, 2000). Emerging literature has underscored the importance of expanding the traditional TAM framework by incorporating additional constructs such as perceived trust, perceived enjoyment, and system quality—variables increasingly recognized as critical for understanding post-adoption behavior (Xu et al., 2022; He et al., 2023). These constructs reflect users’ deeper emotional and cognitive evaluations of platform experiences, which significantly influence adoption and sustained engagement—particularly in educational contexts where long-term system interaction is essential for academic success.

To address these gaps, many studies have attempted to enhance TAM’s explanatory power by introducing new variables or extending the model. For instance, Liu et al. (2024) added e-service quality as an exogenous factor to TAM, demonstrating that e-service quality positively influences users’ perceived usefulness and ease of use, thereby addressing TAM’s limited focus on external factors. Additionally, Esteban-Millat et al. (2018) introduced users’ flow experience as an emotional variable, finding that flow experience significantly positively affects users’ attitudes and behavioral intentions, thus overcoming TAM’s rational behavior assumptions. In the educational domain, TAM has been further extended to understand technology adoption better. For example, Niu and Wu (2022) integrated the Technology Acceptance Model (TAM) with the Task-Technology Fit (TTF) model to construct a framework explaining factors influencing creativity in online learning environments, particularly for vocational college students. Similarly, Velicia-Martin et al. (2021) highlighted that while TAM has value in explaining college students’ acceptance of mobile learning, its limitations lie in its insufficient consideration of emotional factors and long-term usage behaviors.

Recent advancements in technology have introduced intelligent learning platforms that leverage big data, artificial intelligence (AI), and cloud computing to enhance educational efficiency and effectiveness. These platforms provide personalized learning experiences, improving student engagement and interaction (Zhu et al., 2020). However, despite their potential, challenges such as inconsistent platform management and insufficient data integration persist, hindering their effectiveness (He et al., 2023). While TAM provides a robust theoretical framework for understanding technology acceptance and use, its application in educational contexts may fall short of explaining sustained user engagement. Therefore, future research should further extend TAM by incorporating variables such as perceived trust, perceived enjoyment, and system quality to provide a more comprehensive understanding of user behavior in digital learning platforms. This line of inquiry not only contributes to theoretical refinement but also holds practical value for developers and educators aiming to improve the learning experience and student retention. This approach will offer practical strategies for platform optimization to enhance user retention and learning outcomes. Accordingly, this study is guided by the following research questions:

1. What are the most salient predictors of students’ continued use of innovative learning platforms?
2. How do perceived trust, perceived enjoyment, and system quality influence students’ perceptions of usefulness and ease of use?
3. To what extent do these variables affect long-term continuance intention?

Data were collected from a large-scale sample of 782 higher vocational college students in China to examine these questions empirically. Structural Equation Modeling (SEM) was employed to test the proposed relationships among key constructs. By integrating cognitive (e.g., perceived usefulness, perceived ease of use) and affective (e.g., trust, enjoyment) dimensions, the proposed model offers a comprehensive perspective on the psychological and technological mechanisms that underlie continued usage in innovative learning environments.

The remainder of this paper is structured as follows: Section 2 provides a review of relevant literature on TAM and its extensions; Section 3 details the research methodology, including survey design and analytical procedures; Section 4 presents the empirical findings; Section 5 discusses the theoretical contributions and practical implications; and Section 6 concludes with study limitations and suggestions for future research.

## 2 Literature review

### 2.1 Online learning platforms and adoption challenges

Online learning platforms have become essential components of contemporary higher education, offering increased flexibility, accessibility, and interactive learning opportunities (Al-Adwan et al., 2023; Pan et al., 2024). These platforms, powered by big data analytics, artificial intelligence (AI), and cloud-based technologies, aim to enhance pedagogical effectiveness and promote long-term learner engagement (Xu et al., 2022). Prior empirical studies underscore the critical roles of system functionality, content organization, and navigational ease in shaping student retention and satisfaction within digital learning environments (Chen et al., 2021). Moreover, user trust—particularly regarding the credibility of content and the security of the platform—has emerged as a key determinant of users’ continued engagement (Bhati et al., 2023; He et al., 2023). However, while these functional and security-related features are foundational, they alone are insufficient to account for sustained user behavior—especially in more demanding digital learning contexts.

Beyond these functional elements, increasing scholarly attention has been directed toward the affective and motivational dimensions of user experience. Constructs such as perceived enjoyment have been consistently linked to sustained platform use (Chugh et al., 2023). In fact, both perceived enjoyment and user satisfaction are now widely acknowledged as indispensable for maintaining long-term engagement in online learning—particularly in environments that are cognitively and emotionally demanding. The abrupt transition to online learning during the COVID-19 pandemic further highlighted the complexity of sustaining digital engagement. As Mora-Cruz et al. (2023) demonstrated, both educators and students encountered considerable challenges in adapting to fully digital formats, revealing the dual importance of technical usability and emotional adaptability. This perspective is further reinforced by Ficapal-Cusí et al. (2024), who examined the roles of user intention, enjoyment, and habitual use in influencing students’ wellbeing and long-term interaction with e-learning platforms. Their findings suggest that emotional engagement and routine platform usage are critical in cultivating positive learning experiences. These dynamics may be particularly salient for vocational education students, who often lack prior exposure to advanced digital tools and may face amplified stress during rapid transitions. Collectively, these studies support the broader argument that cognitive and affective mechanisms jointly shape behavioral engagement in digital learning (Chugh et al., 2023).

However, despite the expanding body of research on online learning within general higher education, vocational education contexts remain underexplored. Vocational students often engage with digital platforms under compulsory or institutionalized conditions and may exhibit distinctive patterns of motivation, satisfaction, and continuance intention. Existing empirical studies have highlighted the importance of practical relevance, interface simplicity, and emotional support in sustaining engagement in such contexts (Zhang and Chen, 2021; Chi, 2019). For instance, Zhang and Chen (2021) applied sentiment analysis to explore vocational students’ emotional responses. They found that satisfaction and frustration frequently coexisted—particularly in situations marked by low digital literacy or system overload. Chi (2019) further emphasized that while trust and usability are foundational, they may be insufficient to ensure satisfaction if students lack adequate psychological support. Inadequate emotional scaffolding—such as unclear guidance, lack of interaction, or perceived neglect—can erode the positive effects of trust, leading to disengagement even in technically competent platforms.

These observations suggest that the factors shaping technology adoption in vocational education may differ substantially in both magnitude and mechanism from those in traditional higher education. Therefore, this study seeks to address this research gap through a context-specific lens. By integrating both cognitive (e.g., perceived usefulness and ease of use) and emotional (e.g., trust and enjoyment) constructs into an extended TAM-ISCM framework, this study aims to provide a nuanced understanding of the determinants of sustained engagement with innovative learning platforms among vocational learners. Furthermore, by testing the interactions among trust, enjoyment, and satisfaction, this research seeks to uncover the mechanisms through which psychological and experiential factors influence continuance intention in institutionally mandated learning environments.

### 2.2 The technology acceptance model (TAM) and its extensions

The integration of emotional and experiential dimensions into the Technology Acceptance Model (TAM) has garnered increasing scholarly attention, particularly within digital learning environments. While foundational cognitive constructs—such as perceived usefulness (PU) and perceived ease of use (PEOU)—remain central to technology adoption theory, growing evidence suggests they alone are insufficient to explain user behavior, particularly in complex educational settings fully. Learners’ affective responses, including perceived enjoyment and satisfaction, play a critical role in shaping both initial acceptance and long-term engagement (Mora-Cruz et al., 2023). These findings have prompted researchers to expand TAM by incorporating motivational and emotional elements to capture better the full spectrum of user experience (Esteban-Millat et al., 2018; Oyman et al., 2022). For instance, Ficapal-Cusí et al. (2024) emphasized the impact of user intention, emotional engagement, and habitual use on students’ wellbeing and sustained interaction with e-learning platforms. Collectively, these developments reflect the evolution of TAM into a more comprehensive and context-sensitive framework capable of addressing the complexities of modern educational adoption behaviors.

#### 2.2.1 Emotional and experiential dimensions

Originally proposed by Davis (1989), TAM was initially grounded in cognitive antecedents—most notably PU and PEOU—to explain early-stage adoption. These constructs have been widely validated across various domains, particularly in higher education (Venkatesh and Bala, 2008). However, recent research increasingly points to the importance of emotional and experiential dimensions in sustaining long-term technology usage. To extend TAM’s explanatory scope, scholars have incorporated constructs such as perceived trust, enjoyment, flow experience, and system quality, which capture affective, immersive, and subjective user experiences.

For example, Esteban-Millat et al. (2018) integrated the concept of “flow” into TAM, demonstrating that immersive experiences significantly enhance PU, PEOU, and usage intentions. Similarly, Oyman et al. (2022) found that emotional engagement indirectly strengthened continuance intention via increased perceived usefulness, particularly in asynchronous learning environments. These insights suggest that TAM’s traditional reliance on rational evaluation may be inadequate in educational settings where emotional investment, autonomy, and user agency are critical to sustained engagement. This is especially pertinent to vocational education contexts, where students’ platform interactions are shaped not only by usability and performance but also by affective satisfaction and contextual relevance.

#### 2.2.2 TAM and other model integration

Building upon TAM’s foundational structure, researchers have increasingly sought to merge it with complementary theoretical models to better account for the nuanced mechanisms underlying continued technology use. One prominent extension involves the Information Systems Continuance Model (ISCM), which emphasizes user satisfaction and expectation confirmation as the primary determinants of sustained engagement (Bhattacherjee, 2001). The integrated TAM-ISCM approach provides a multidimensional lens through which both adoption and retention can be analyzed, especially in digitally mediated learning environments. Xu et al. (2022), for instance, applied a TAM-ISCM framework in the context of e-learning and found that system quality—defined by usability, interface design, and platform reliability—directly influenced satisfaction and perceived usefulness. Furthermore, trust in the platform’s security and technical integrity emerged as a key driver of satisfaction and user loyalty. These findings underscore the importance of aligning functional performance with user expectations to foster sustained platform engagement.

In addition, scholars have extended TAM through psychological theories such as Self-Determination Theory (SDT), which emphasizes autonomy, competence, and relatedness as intrinsic motivational drivers (Deci and Ryan, 1985). Venkatesh et al. (2003) demonstrated that perceived autonomy and enjoyment significantly enhanced PU, satisfaction, and continuance intention, mainly when learners had control over their learning pace and content. Another influential integration involves the Task-Technology Fit (TTF) model, which posits that user satisfaction and performance are optimized when technological features align with specific task requirements (Goodhue and Thompson, 1995). Studies by Chen et al. (2021) and Liu et al. (2024) showed that when e-learning platforms supported self-directed learning, task customization, and content relevance, students were more likely to perceive the platform as valid and continue its usage. This alignment reduces cognitive friction and enhances the overall user experience.

Taken together, these model integrations illustrate the dynamic evolution of TAM into a more holistic framework. By incorporating affective, contextual, and motivational factors, TAM extensions offer a robust basis for analyzing technology adoption—particularly in vocational education, where continued platform use is often mandatory and emotionally mediated.

#### 2.2.3 Cross-disciplinary applications of TAM: a summary of the literature

The Technology Acceptance Model (TAM) has demonstrated notable conceptual adaptability across a wide range of domains beyond its original focus in educational contexts. As illustrated in Table 1, scholars have extended TAM by incorporating domain-specific constructs, thereby enhancing its explanatory capacity in diverse sectors such as tourism, business management, non-profit organizations, healthcare, virtual learning, and personal wellbeing. These applications collectively underscore TAM’s relevance across both public and private domains where technology adoption is behaviorally and contextually nuanced.

**Table 1:** Examples of TAM-based studies in various fields.

Field	Study	Key constructs and focus
Tourism	Palos-Sánchez et al. (2017)	Examines user intention and privacy concerns in the context of location-based services.
Management	Palos-Sánchez et al. (2017)	Investigate cloud computing (SaaS) adoption as a strategic technology in businesses.
Non-profit organizations	Saura et al. (2020)	It focuses on digital platform acceptance for NGO projects and exploring volunteer behavior.
Healthcare	Velicia-Martin et al. (2021)	Research on COVID-19 tracing app acceptance, integrating the TAM framework with health behavior.
Higher education	Wang et al. (2022)	Applies TAM to MOOC design, integrating user motivation and autonomy to predict engagement and learning effectiveness.
Virtual learning	Xie et al. (2022)	Applies TAM in VR-based learning environments, emphasizing interaction quality and immersion.
Fitness and wellbeing	Yuan et al. (2015)	Examines fitness app adoption, adding trust and social influence to traditional TAM.

The Technology Acceptance Model (TAM) has evolved into a highly adaptable theoretical framework, demonstrating applicability across a wide range of disciplines beyond its foundational use in educational technology. As outlined in Table 1, researchers have extended TAM to various sectors—including tourism, business management, healthcare, non-profit organizations, fitness and wellness, as well as immersive virtual learning—by integrating domain-relevant constructs. These modifications have enriched the model’s explanatory power, allowing it to account for sector-specific behavioral patterns and contextual contingencies (Palos-Sánchez et al. 2017; Velicia-Martin et al., 2021; Yuan et al., 2015; Xie et al., 2022). In the tourism and healthcare domains, for instance, privacy concerns and trust have been identified as pivotal in influencing user adoption decisions, particularly in environments where sensitive personal data is routinely processed (Palos-Sánchez et al. 2017; Velicia-Martin et al., 2021). In the context of fitness and wellbeing, studies have underscored the significance of social influence and community support as salient determinants of both adoption and continued engagement with digital health platforms (Yuan et al., 2015). Meanwhile, within higher education and virtual learning environments, TAM has been enriched through the incorporation of psychological constructs such as self-efficacy, perceived immersion, and subjective norms, thereby offering more nuanced explanations of learner engagement—especially in the face of emerging technologies like MOOCs and metaverse-based platforms (Wang et al., 2022; Xie et al., 2022).

Taken together, these cross-sectoral applications underscore the theoretical robustness and contextual elasticity of TAM. They affirm that the model’s core tenets—perceived usefulness and perceived ease of use—can be meaningfully extended through the addition of field-specific variables to capture more complex adoption dynamics. Moreover, these adaptations frequently align with broader psychological and technological frameworks, such as Self-Determination Theory (SDT) and Task-Technology Fit (TTF), thus enabling a multidimensional understanding of user behavior. This ongoing evolution of TAM continues to support its relevance in analyzing technology acceptance and sustained use across diverse applied contexts.

### 2.3 Research gap

Despite the growing body of research on online learning platforms and the widespread application of the Technology Acceptance Model (TAM) across various domains, notable research gaps persist—particularly in the context of vocational college education. Existing studies have predominantly focused on short-term adoption intentions, providing limited insight into the psychological and behavioral mechanisms that underpin sustained user engagement over time (Bhattacherjee, 2001; Xu et al., 2022). While constructs such as trust, enjoyment, and system quality have each been explored in isolation, these efforts remain fragmented. There is a lack of integrative frameworks that explain how emotional, cognitive, and technical dimensions interact dynamically to influence long-term usage behavior.

This limitation is especially salient in educational contexts, where learner autonomy, intrinsic motivation, and platform usability increasingly shape digital engagement patterns (Venkatesh et al., 2003; Chen et al., 2021). Furthermore, empirical extensions of TAM have concentrated mainly on commercial, healthcare, or general higher education settings, with insufficient attention paid to vocational education—a domain characterized by distinct learner profiles, skill-based instruction, and frequent institutional mandates for platform use. Vocational students, in particular, tend to exhibit unique patterns of technology interaction due to their emphasis on practical learning, variable digital competence, and high dependence on institutional e-learning platforms. These contextual features may fundamentally alter how key TAM constructs operate in such environments. Without targeted investigations, models derived from university contexts risk overlooking the nuanced realities of vocational learners.

To bridge these gaps, the present study develops and empirically tests an extended TAM framework that integrates both cognitive (e.g., perceived usefulness, perceived ease of use) and affective (e.g., trust, enjoyment) constructs to explain the continued use of online learning platforms. Specifically, the model explores how perceived trust, system quality, and enjoyment jointly influence satisfaction and continuance intention. Drawing on data collected from a large-scale survey of vocational college students in China and analyzed using Structural Equation Modeling (SEM), this study contributes not only practical guidance for improving digital learning design but also theoretical refinement of TAM applications in underexplored, mandatory-use educational contexts.

## 3 Methodology

### 3.1 Research design

This study adopts a realist ontological stance, positing that an objective reality exists independently of individual perceptions and can be empirically examined. Anchored in a positivist epistemological framework, the study employs quantitative methods to investigate factors influencing the continued use of innovative learning platforms in higher education. A structured, hypothesis-driven approach explores the interplay among key variables such as perceived usefulness, perceived ease of use, perceived trust, and continuance intention.

**Figure 1:**
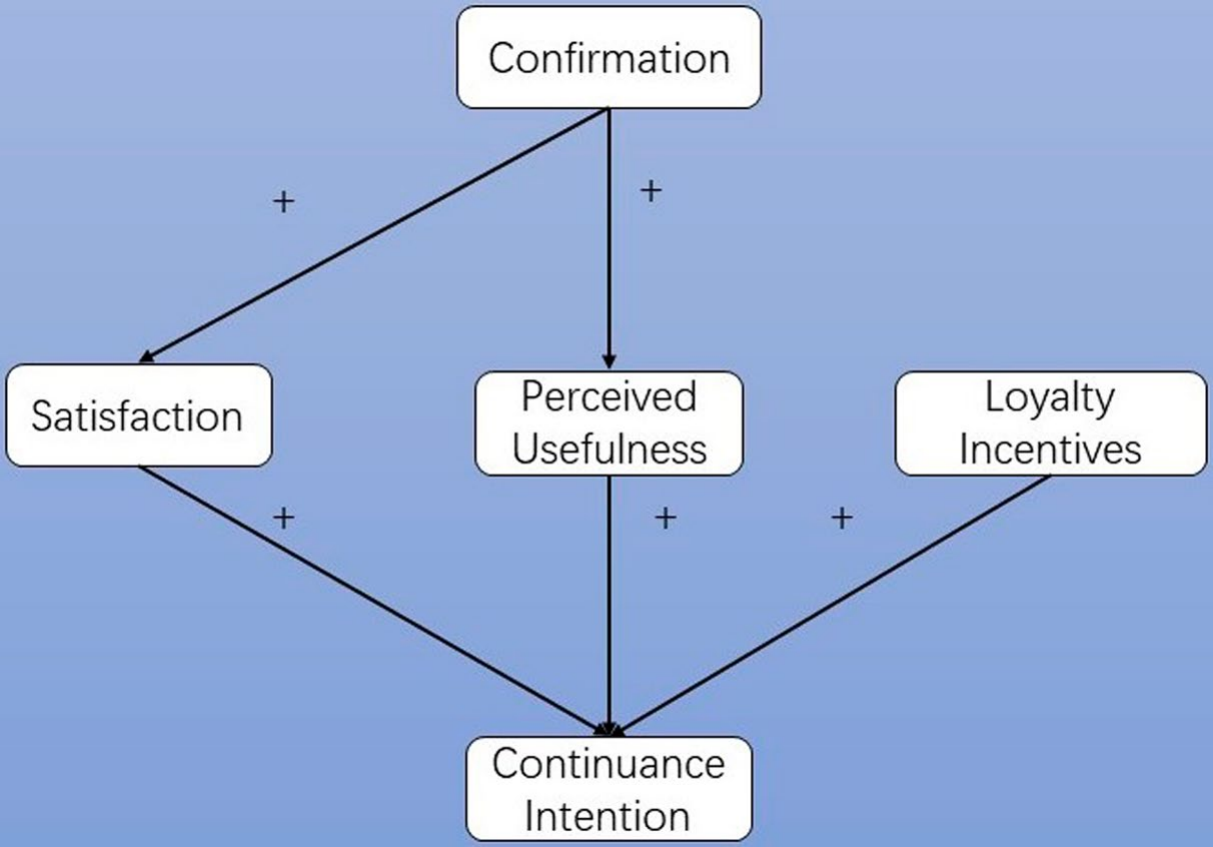
Information systems sustainability model redrawn based on Bhattacherjee.

Building upon the foundational Technology Acceptance Model (TAM), this research extends the theoretical framework by incorporating constructs particularly salient in digital learning contexts: perceived trust, enjoyment, and user satisfaction. These additions reflect technology usage’s emotional and experiential dimensions, which are critical in post-adoption behavior. Bhattacherjee’s Information System Continuance Model (ISCM) further informs the conceptual model, which introduces confirmation and satisfaction as antecedents to continuance intention. Figure 1 illustrates the theoretical model, adapted from Bhattacherjee’s ISCM, tailored to the specific dynamics of innovative learning platforms in higher education. This model forms the conceptual basis for the hypotheses presented in the following section.

### 3.2 Questionnaire design

A structured questionnaire was developed using measurement items adapted from previously validated instruments to validate the proposed model empirically. All items were measured on a five-point Likert scale ranging from 1 (strongly disagree) to 5 (strongly agree), ensuring comparability and reliability across constructs.

#### 3.2.1 Questionnaire structure

The questionnaire comprised three main sections. The first section captured demographic information, including gender, political affiliation, academic discipline, and year of study. The second section assessed users’ behavioral patterns and attitudes toward innovative learning platforms, such as frequency of use and perceived usefulness. The third section measured latent constructs derived from the research model, including confirmation (expectation vs. reality), perceived ease of use, usefulness, trust, satisfaction, enjoyment, system quality, information quality, and continuance intention.

#### 3.2.2 Measurement items and scale sources

The survey was grounded in an extended TAM framework, incorporating additional constructs that reflect technological performance and psychological engagement. Items were drawn from established sources to ensure construct validity. Perceived usefulness and ease of use items were based on Davis (1989), while confirmation and satisfaction items followed the Expectation-Confirmation Model (Bhattacherjee, 2001; Oliver, 1980). Perceived trust was measured using items from Tan (2019), and perceived enjoyment was adapted from Venkatesh et al. (2003) and Tsang (2004). System and information quality dimensions followed the DeLone and McLean (1992) model. Table 2 presents the construct definitions and corresponding measurement items. Each construct was operationalized using three to four items, capturing core aspects of user attitudes, system perceptions, and behavioral intentions within digital learning environments. These validated items ensured the measurement model’s content reliability and construct validity.

**Table 2:** Questionnaire item design for measuring constructs in the extended TAM framework.

Construct	Item code	Questionnaire item	Source
Perceived usefulness	PU1	Using the smart learning platform can improve my learning efficiency.	Bhattacherjee (2001)
	PU2	Using the platform can improve the quality of my learning.	
	PU3	I can find practical knowledge and information related to fundamental theory.	
	PU4	The content of the platform is strictly controlled and very useful.	
Perceived ease of use	PEU1	It is easy for me to use the platform without external help.	Davis (1989), Moon and Kim (2001), and Hong et al. (2006)
	PEU2	The interactive interface of the platform is straightforward to understand.	
	PEU3	It is very convenient to use the platform to learn relevant resources.	
	PEU4	The platform is simple and easy to use, with fast operation.	
Perceived trust	PT1	The platform is trustworthy.	Tan (2019)
	PT2	I believe the platform will not leak my private information.	
	PT3	The learning materials provided are authoritative and reliable.	
Confirmation	CON1	The experience and gains of using the platform to learn exceed my expectations.	Bhattacherjee (2001)
	CON2	The platform experience is higher than expected before using it.	
	CON3	The content and quality control surpass my initial expectations.	
Satisfaction	SAT1	I am satisfied with the learning resources and activities provided by the platform.	Bhattacherjee (2001) and Oliver (1980)
	SAT2	I am satisfied with the functional modules of the platform.	
	SAT3	I am satisfied with the learning experience on the platform.	
	SAT4	Overall, I am delighted with my use of the platform.	
Perceived enjoyment	PE1	Using the platform makes me feel more relaxed, learn efficiently, and be happy.	Davis (1989) and Tsang (2004)
	PE2	The platform offers exciting content like micro-videos, case studies, and e-books.	
	PE3	Using the platform is an enjoyable and exciting process.	
Platform quality	SYS1	The platform’s response speed is fast, allowing smooth use.	DeLone and McLean (1992)
	SYS2	Each function’s design is perfect and stable in operation.	
	SYS3	The interface layout is user-friendly and easy to use for novices.	
Information quality	INF1	The platform provides sufficient content with quick updates.	DeLone and McLean (1992)
	INF2	The platform’s learning resources are strictly controlled and reliable.	
	INF3	The platform’s content attracts my attention.	
Continued use intention	CI1	I will continue to use the platform to study.	Bhattacherjee (2001)
	CI2	If possible, I will frequently use the platform.	
	CI3	I will recommend the platform to others.	

**Figure 2:**
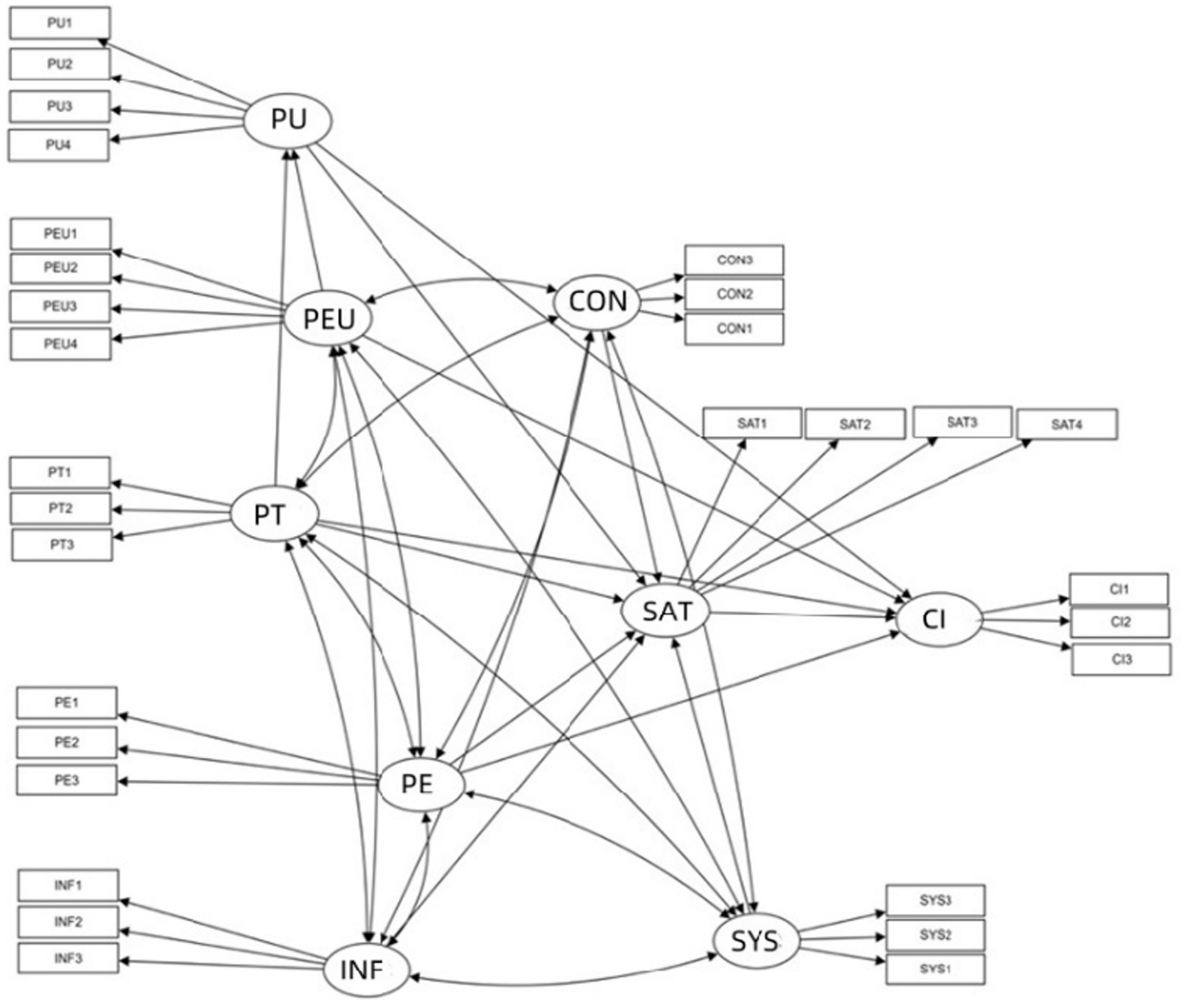
Research model on continuous use of innovative learning platform users.

Figure 2 presents the extended research model incorporating TAM and Expectation-Confirmation Theory elements. In addition to the traditional TAM components (PU and PEOU), the model integrates PT, PE, SYS, INF, and SAT to provide a more comprehensive understanding of sustained user engagement with innovative learning platforms. These added dimensions reflect the increasingly complex interplay of trust, affective experience, and technical performance in influencing user continuance intention. This integrative model offers a nuanced framework for evaluating technology adoption in educational settings, especially where long-term engagement is crucial. It acknowledges that beyond usability and functionality, emotional and contextual variables such as trust, enjoyment, and content reliability are essential to promoting the consistent use of educational technologies.

### 3.3 Hypotheses development

This study proposes its hypotheses by extending two seminal theoretical frameworks: the Technology Acceptance Model (TAM; Davis, 1989) and the Information System Continuance Model (ISCM; Bhattacherjee, 2001). While TAM primarily captures users’ pre-adoption cognitive evaluations—specifically perceived usefulness and perceived ease of use—ISCM incorporates post-adoption variables such as expectation confirmation and user satisfaction to explain sustained usage. To build a more comprehensive understanding of long-term engagement with educational technologies, this study further integrates affective and contextual dimensions, namely perceived trust, perceived enjoyment, and system quality, into the TAM-ISCM framework. These constructs are particularly salient in digital learning environments where user engagement is shaped not only by utility and usability but also by affective experience, perceived reliability, and system responsiveness. Each hypothesis is developed based on well-established theoretical reasoning and supported by empirical evidence from prior research.

- Perceived usefulness (PU)

Perceived usefulness (PU) refers to the extent to which students believe that using an online learning platform will enhance their academic performance. Within the ISCM framework, PU has been widely identified as a key cognitive predictor of satisfaction (Bhattacherjee, 2001). Empirical studies across both general and educational technology contexts support this relationship (Venkatesh and Bala, 2008; Al-Adwan et al., 2023). Liu (2013) also validated the influence of PU in the Chinese higher education setting. Based on these findings, the following hypothesis is proposed:

*H1*: Perceived usefulness positively influences user satisfaction.

PU also plays a central role in TAM and ISCM as a determinant of continued usage intention (Bhattacherjee, 2001; Venkatesh and Bala, 2008). Students are more likely to maintain the use of platforms they perceive as helpful for academic success, as evidenced by Zhou et al. (2022) and Liu (2013). Therefore, the following hypothesis is proposed:

*H2*: Perceived usefulness positively influences continuance intention.

- Expectation confirmation (CON)

According to the Expectation-Confirmation Theory (Oliver, 1980), user satisfaction arises when actual performance meets or exceeds prior expectations. This concept is foundational in ISCM and has been widely applied to digital education (Bhattacherjee, 2001; Liu, 2013). Based on these theoretical foundations, the following hypothesis is proposed:

*H3*: Expectation confirmation positively influences user satisfaction.

Furthermore, when expectations are met, users tend to revise upward their evaluations of the platform’s utility. This relationship is supported in both information systems and e-learning contexts (Bhattacherjee, 2001; Zhou et al., 2022). Thus, the following hypothesis is:

*H4*: Expectation confirmation positively influences perceived usefulness.

- User satisfaction (SAT)

User satisfaction is a central post-adoption construct in ISCM. It reflects the extent to which the platform meets learners’ academic and psychological needs. High satisfaction has been linked to more assertive continued usage across many studies (Bhattacherjee, 2001; Zhou et al., 2022; Liu, 2013). Therefore, the following hypothesis is proposed:

*H5*: User satisfaction positively influences continuance intention.

- Perceived ease of use (PEOU)

TAM posits that platforms perceived as easy to use are more likely to be evaluated as applicable, as ease of use reduces effort and cognitive burden (Davis, 1989). This relationship has been consistently verified in education technology research, especially in contexts with complex tasks (Venkatesh and Davis, 2000; Gefen and Straub, 2000). Accordingly, the following hypothesis is proposed:

*H6*: Perceived ease of use positively influences perceived usefulness.

In addition to this indirect path through PU, PEOU may also directly contribute to continuance intention by lowering perceived barriers to engagement. In self-directed and digital learning contexts, ease of navigation is a known enabler of sustained engagement (Sánchez-Prieto et al., 2016; Chugh et al., 2023). Thus, the following hypothesis is:

*H7*: Perceived ease of use positively influences continuance intention.

- Perceived trust (PT)

Trust in the platform—its stability, data protection, and content credibility—can significantly shape students’ perceptions of its academic value. Prior research has established that trust increases perceived usefulness (Mustafa and Garcia, 2021; Bhati et al., 2023). Accordingly, the following hypothesis is formulated:

*H8*: Perceived trust positively influences perceived usefulness.

Trust also contributes to a psychologically safe and satisfying user experience, especially in institutional contexts where ethical and functional expectations are high (Chen et al., 2021; He et al., 2023). Therefore, the following hypothesis is:

*H9*: Perceived trust positively influences user satisfaction.

Moreover, trust can act as a psychological anchor that sustains long-term usage. When students feel confident in the platform’s reliability and privacy, they are more likely to continue using it (Bhattacherjee, 2001; Tan, 2019). Based on this rationale, the following hypothesis is proposed:

*H10*: Perceived trust positively influences continuance intention.

- Perceived enjoyment (PE)

Perceived enjoyment—rooted in Self-Determination Theory (SDT)—is a strong predictor of voluntary and sustained technology use. When users find the platform engaging, their intrinsic motivation increases (Deci and Ryan, 1985; Venkatesh et al., 2003), based on these principles, the following hypothesis is proposed:

*H11*: Perceived enjoyment positively influences continuance intention.

Beyond continuance intention, enjoyment has also been shown to enhance satisfaction, even in compulsory learning environments directly. Studies confirm a strong correlation between enjoyable digital experiences and satisfaction (Kashive and Mohite, 2023; Nguyen, 2022; Huang and Liu, 2024). Therefore, the following hypothesis is:

*H12*: Perceived enjoyment positively influences user satisfaction.

- System quality (SYS)

System quality—defined by interface design, technical reliability, and responsiveness—affects user satisfaction by minimizing frustration and enhancing the learning experience. This is a key dimension in the Information Systems Success Model (DeLone and McLean, 1992) and remains relevant in digital education (Al-Adwan et al., 2023; Zhou et al., 2022). Based on this theoretical foundation, the final hypothesis is proposed:

*H13*: System quality positively influences user satisfaction.

### 3.4 Sampling, data collection, and analysis methods

To ensure representative coverage of the target population, this study employed a stratified sampling strategy. A total of 865 questionnaires were distributed across more than 20 higher vocational colleges in various regions of China. The participating institutions were selected based on criteria such as geographic location, academic discipline diversity, and institutional size in order to capture a broad spectrum of student experiences. After rigorous data screening—including the removal of incomplete or inconsistent responses—782 valid questionnaires were retained for analysis. The retained sample maintained sufficient demographic and disciplinary diversity to support generalizable inferences about vocational college students. Participants were full-time students enrolled in vocational education programs spanning multiple academic domains. These included humanities and social sciences (e.g., literature, education, economics, and management), science and engineering (e.g., natural sciences, engineering, agriculture, and medicine), as well as arts-related fields. The gender distribution was relatively balanced, with 44.28% identifying as male and 55.72% as female. In terms of the academic year, the sample reflected the typical three-year structure of Chinese vocational education: 27.37% were first-year students, 45.01% were second-year students, 23.21% were third-year students, and 4.47% were categorized as “others.” This demographic diversity strengthens the interpretability of subsequent behavioral analyses. A detailed breakdown is provided in Table 3.

**Table 3:** Demographic distribution of the sample.

Category	Group	Frequency	Percentage
Gender	Male	346	44.28%
	Female	436	55.72%
Grade	First-year	214	27.37%
	Second-year	352	45.01%
	Third-year	182	23.21%
	Others	34	4.47%
Major category	Humanities and Social Sciences	288	36.82%
	Science and Engineering	313	40.03%
	Others (e.g., Art-related disciplines)	181	23.14%

Following the sample recruitment, data collection was conducted using the Wenjuanxing platform, a widely adopted online survey tool in China. Questionnaires were disseminated via institutional communication channels, including campus-wide email systems and official learning management platforms. All participants were informed of the study’s objectives and assured of anonymity and confidentiality in compliance with ethical research standards. To ensure data quality, responses exhibiting suspicious patterns—such as identical answers across all items, excessively short completion times, or extensive missing values—were deemed invalid and excluded from the final dataset. After this cleaning process, the dataset remained robust in both size and variability.

A series of statistical analyses were performed to assess the reliability and validity of the measurement model. Internal consistency was evaluated using Cronbach’s alpha in SPSS 26.0. Confirmatory Factor Analysis (CFA) was conducted in AMOS 24.0 to assess construct validity. Furthermore, Structural Equation Modeling (SEM) was applied to test the hypothesized relationships among the latent constructs within the extended TAM framework. Covariance-based SEM (CB-SEM) was selected over alternatives such as Partial Least Squares SEM (PLS-SEM) and Bayesian SEM due to the study’s large sample size and its confirmatory, theory-driven nature. While emerging hybrid approaches—such as integrating Artificial Neural Networks (ANN) with SEM—show promise in modeling complex, non-linear relationships, they were deemed beyond the scope of the current study. CB-SEM was, therefore, chosen as the most appropriate analytical method for rigorously testing theoretically grounded hypotheses within this research context.

## 4 Results

### 4.1 Validity and reliability analysis

A series of diagnostic tests were conducted to assess the psychometric soundness of the measurement instrument. Cronbach’s alpha for the overall scale was 0.990, indicating excellent internal consistency across the 30 measurement items (see Table 4). The Kaiser-Meyer-Olkin (KMO) measure of sampling adequacy was 0.982—well above the recommended threshold of 0.6—suggesting suitability for factor analysis. Additionally, Bartlett’s Test of Sphericity yielded a chi-square value of 40,038.048 (df = 435, *p* < 0.001), confirming the factorability of the correlation matrix (Table 5). Following confirmation of sampling adequacy and matrix factorability, PCA was conducted to explore the underlying component structure of the instrument. Principal Component Analysis (PCA) showed that the first two components accounted for 82.805% of the total variance (Table 6), demonstrating that the instrument captures a substantial proportion of variance among the latent constructs.

**Table 4:** Cronbach’s alpha.

Reliability statistics	Alpha value	Items
Cronbach’s Alpha	0.99	30

**Table 5:** KMO and Bartlett’s test.

KMO measure of sampling adequacy	0.982
Approx. chi-square	40038.048
Degrees of freedom	435
Significance level (*p*-value)	0.000

**Table 6:** Total variance explained.

Component	Initial eigenvalues	Extraction sums of squared loadings	Rotation sums of squared loadings
Total	23.574	23.574	15.813
% of Variance	78.580	78.580	52.712
Cumulative %	78.580	78.580	52.712

### 4.2 Measurement model assessment

The reliability and validity of the latent constructs were further evaluated using SPSS 25.0 and AMOS 24.0. Cronbach’s alpha and Composite Reliability (CR) for all constructs exceeded recommended benchmarks of 0.7 and 0.9, respectively, confirming high internal consistency (see Table 7). Convergent validity was supported by Average Variance Extracted (AVE) values above 0.5 and significant factor loadings (all *p* < 0.001), which were consistently >0.7. Construct validity was examined using the criteria set forth by Fornell and Larcker (1981). High standardized loadings, CR values above 0.9, and AVE values above 0.5 collectively affirmed the robustness of the measurement model. These indicators validate the measurement model’s construct reliability and convergent validity.

**Table 7:** Reliability and validity analysis.

Variable	Item	Unstd.	S.E	*P*	Std.	SMC	CR	AVE	Cronbach’s α
PEU	PEU2	1.000			0.905	0.819	0.959	0.888	0.821
	PEU3	1.043	0.022	***	0.960	0.922			
	PEU4	1.037	0.021	***	0.960	0.922			
PU	PU1	1.000			0.925	0.856	0.947	0.857	0.825
	PU2	1.035	0.021	***	0.963	0.927			
	PU4	0.957	0.023	***	0.888	0.789			
CON	CON1	1.000			0.970	0.941	0.976	0.932	0.855
	CON2	0.993	0.014	***	0.965	0.931			
	CON3	0.999	0.014	***	0.962	0.925			
SAT	SAT1	1.000			0.962	0.925	0.972	0.921	0.869
	SAT2	1.013	0.015	***	0.966	0.933			
	SAT4	0.991	0.016	***	0.951	0.904			
PT	PT1	1.000			0.923	0.852	0.936	0.831	0.814
	PT2	1.058	0.028	***	0.881	0.776			
	PT3	1.025	0.024	***	0.930	0.865			
SYS	SYS1	1.000			0.936	0.876	0.958	0.884	0.803
	SYS2	0.983	0.019	***	0.943	0.889			
	SYS3	0.964	0.019	***	0.942	0.887			
INF	INF1	1.000			0.952	0.906	0.956	0.879	0.823
	INF2	0.977	0.020	***	0.923	0.852			
	INF3	1.011	0.019	***	0.938	0.880			
CI	CI1	1.000			0.952	0.906	0.968	0.911	0.904
	CI2	1.005	0.017	***	0.958	0.918			
	CI3	1.017	0.017	***	0.953	0.908			
PE	PE1	1.000			0.964	0.929	0.965	0.902	0.815
	PE2	0.938	0.017	***	0.935	0.874			
	PE3	0.982	0.016	***	0.950	0.903			

### 4.3 Structural model fit and hypothesis testing

Structural Equation Modeling (SEM) was employed to test the proposed hypotheses and assess the overall model fit. Fit indices yielded satisfactory values: CMIN/DF = 3.882, GFI = 0.884, AGFI = 0.853, CFI = 0.972, TLI = 0.968, and IFI = 0.972—all meeting or exceeding standard thresholds (Table 8). These results indicate an acceptable fit between the hypothesized model and the empirical data.

**Table 8:** Model fitting index.

Fit index	CMIN/DF	GFI	AGFI	CFI	TAG	IF
Suggested value	<5	>0.8	>0.8	>0.9	>0.9	>0.9
This model value	3.882	0.884	0.853	0.972	0.968	0.972

**Figure 3:**
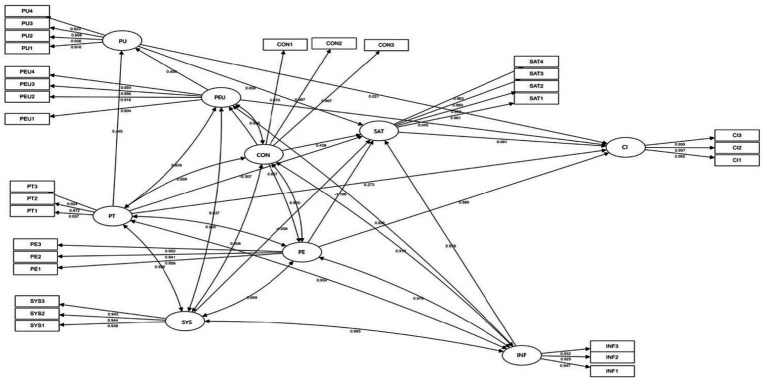
Path confections of factors influencing continued use of innovative learning platform users.

Figure 3 displays the standardized path coefficients among the latent constructs. Positive coefficients represent positive causal effects, while negative coefficients indicate inverse relationships. Statistical significance was determined by *p*-values, with thresholds set at *p* < 0.05. The analysis revealed that perceived trust, enjoyment, and system quality exhibited negative relationships with satisfaction, contradicting hypotheses H9, H12, and H13. This unexpected directionality warrants further exploration in the discussion section, particularly concerning vocational education settings’ unique institutional and psychological dynamics.

Furthermore, hypotheses H1, H2, H4, and H7 were not statistically supported. The lack of significance suggests that these relationships may be context-dependent, moderated or mediated by other psychological or contextual variables not captured in the current model. The empirical data supported the remaining hypotheses, confirming several theoretically grounded relationships and reinforcing the relevance of affective and cognitive dimensions in predicting sustained platform usage.

### 4.4 Model refinement

**Figure 4:**
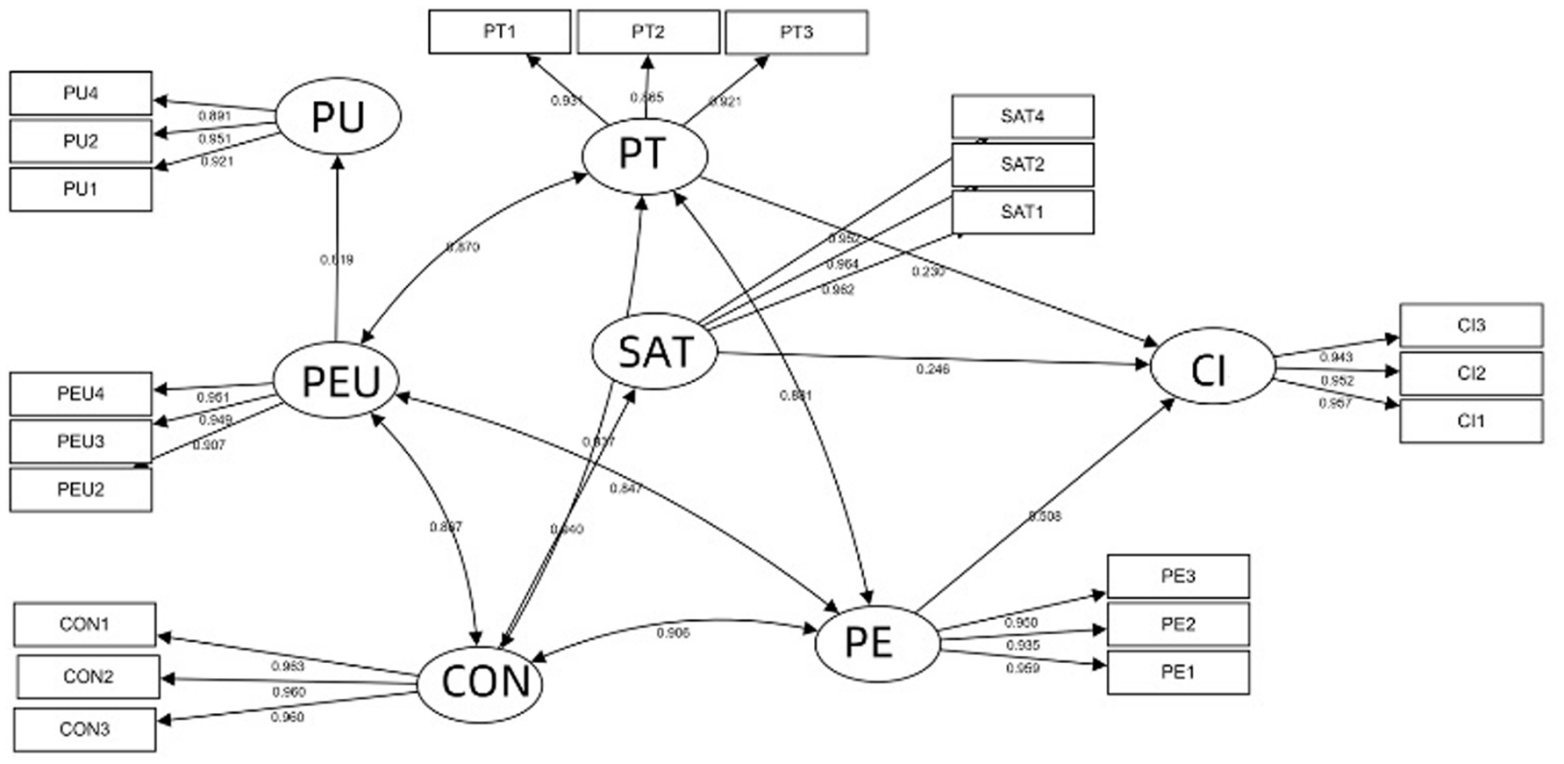
Modified model and path coefficients.

Given the lack of empirical support for several proposed pathways, the model was refined by eliminating non-significant paths, including those from perceived usefulness to satisfaction and continuance intention, perceived ease of use to continuance intention, and satisfaction to continuance intention. After these modifications, the revised model was re-estimated and demonstrated improved parsimony without compromising the overall fit. This refinement enhanced the model’s explanatory power while maintaining an adequate statistical fit. Figure 4 presents the refined structural model, where ellipses denote latent variables, rectangles represent observed indicators, and arrows reflect the hypothesized directional relationships and standardized coefficients. This revised model offers a more empirically grounded representation of the factors influencing sustained engagement with innovative learning platforms among higher vocational education students.

## 5 Discussion

### 5.1 Key findings

This study examined the key determinants of students’ continued engagement with online learning platforms by integrating constructs from the Technology Acceptance Model (TAM) and the Information Systems Continuance Model (ISCM). The empirical results, as presented in Table 9, indicate that both cognitive and emotional evaluations significantly influence continuance intention, reinforcing the dual-path explanatory framework proposed in this study.

**Table 9:** Summary of hypotheses testing results.

Type	Hypothesis ID	Path relationship	Result description
Supported (Positive)	H2	PU → CI	*β* = 0.38, *p* < 0.01
	H3	SAT → CI	*β* = 0.46, *p* < 0.001
	H4	CON → PU	*β* = 0.31, *p* < 0.01
	H5/H6	PEOU → PU	*β* = 0.34, *p* < 0.01
	H8	PT → PU	*β* = 0.29, *p* < 0.01
	H11	PE → CI	*β* = 0.52, *p* < 0.001
Not significant (Unsupported)	H1	PU → SAT	*β* = n.s., *p* > 0.05
	H7	PEOU → CI	*β* = n.s., *p* > 0.05
	H10	PT → CI	*β* = n.s., *p* > 0.05
	H13	SYS → SAT	*β* = 0.05, *p* > 0.05
	H9	PT → SAT	*β* = −0.13, *p* < 0.05
	H12	PE → SAT	*β* = n.s., *p* > 0.05 (negative direction)

Among the predictors, perceived enjoyment emerged as the most influential factor (H11: *β* = 0.52, *p* < 0.001), highlighting the centrality of emotional engagement in sustaining user behavior. This was followed by user satisfaction (H3: *β* = 0.46, *p* < 0.001), perceived usefulness (H2: *β* = 0.38, *p* < 0.01), and perceived ease of use, which indirectly affected continuance intention by significantly enhancing perceived usefulness (H6: *β* = 0.34, *p* < 0.01). Furthermore, expectation confirmation also positively influenced perceived usefulness (H4: *β* = 0.31, *p* < 0.01), underscoring the importance of aligning platform performance with students’ prior expectations. In addition, perceived trust was positively associated with perceived usefulness (H8: *β* = 0.29, *p* < 0.01), suggesting that students who viewed the platform as trustworthy were more likely to regard it as academically valuable. However, two unexpected findings emerged. First, perceived trust exerted a negative effect on satisfaction (H9: *β* = −0.13, *p* < 0.05), contradicting conventional assumptions. Second, system quality was not significantly related to satisfaction (H13: *β* = 0.05, *p* > 0.05), indicating that technical performance alone may be insufficient to enhance satisfaction in specific contexts.

These contradictory results reflect the distinct dynamics of vocational education settings, where platform use is typically mandatory rather than voluntary. In such environments, high levels of trust may elevate expectations, which, if unmet, result in disappointment rather than satisfaction. This highlights the need for a nuanced understanding of how trust and satisfaction interact under institutional constraints. In summary, the findings confirm that both emotional (e.g., enjoyment, satisfaction) and cognitive (e.g., usefulness, trust) factors are integral to explaining continued technology use. At the same time, they reveal that context-specific mechanisms—particularly in mandatory-use environments like vocational colleges—must be considered when extending TAM-based models. These insights contribute to both the theoretical refinement and practical applicability of technology adoption frameworks in underexplored educational settings.

### 5.2 Theoretical interpretation and comparative analysis

This section offers a theoretical interpretation of the empirical findings by classifying the tested hypotheses into three categories: supported (i.e., statistically significant positive relationships), unsupported (i.e., statistically non-significant relationships), and contradictory (i.e., significant negative relationships). Each group is analyzed in light of established theoretical frameworks to elucidate this study’s contributions and highlight context-specific nuances—particularly those emerging from vocational education, where platform usage tends to be mandatory and user expectations may differ from those in general higher education.

#### 5.2.1 Supported hypotheses (positive relationships)

Several hypotheses received empirical support, confirming robust positive associations among key constructs. Most notably, perceived enjoyment demonstrated the most decisive influence on continuance intention (H11: *β* = 0.52, *p* < 0.001), reaffirming Self-Determination Theory (SDT; Deci and Ryan, 1985), which emphasizes the importance of intrinsic motivation for sustaining behavioral engagement. This finding aligns with prior research (e.g., Mora-Cruz et al., 2023; Pan et al., 2024; Ficapal-Cusí et al., 2024), all of which underscore the pivotal role of emotional engagement in fostering long-term digital learning involvement. Within the vocational context, where learners often operate under rigid structures, the capacity of enjoyment to sustain voluntary engagement is particularly noteworthy. Similarly, user satisfaction emerged as a significant predictor of continuance intention (H3: *β* = 0.46, *p* < 0.001), echoing the core propositions of the Information Systems Continuance Model (ISCM) and supported by earlier findings (e.g., Bhattacherjee, 2001; Zhou et al., 2022). The significance of perceived usefulness in predicting continuance intention (H2: *β* = 0.38, *p* < 0.01) further reinforces its central role in the Technology Acceptance Model (TAM), highlighting that students’ perceptions of instrumental value remain a key determinant of platform use, even in mandated settings.

Furthermore, perceived ease of use significantly impacted perceived usefulness (H6: *β* = 0.34, *p* < 0.01), in line with foundational TAM propositions (Davis, 1989). The positive influence of expectation confirmation on perceived usefulness (H4: *β* = 0.31, *p* < 0.01) is consistent with Expectation-Confirmation Theory (Oliver, 1980) and validated by empirical evidence (Liu, 2013; Zhou et al., 2022), suggesting that alignment between prior expectations and experience plays a critical role in shaping perceived utility—especially when system use is institutionally required. Lastly, perceived trust significantly enhanced perceived usefulness (H8: *β* = 0.29, *p* < 0.01), indicating that students who regard a platform as reliable and credible are more inclined to view it as academically beneficial. This supports the notion that trust serves as a cognitive amplifier of perceived value. This effect is particularly relevant in vocational education, where institutional authority shapes expectations and learning trajectories.

#### 5.2.2 Unsupported hypotheses (non-significant relationships)

In contrast, several hypothesized pathways did not reach statistical significance, suggesting that some established TAM and ISCM mechanisms may not function uniformly across educational contexts. For instance, perceived usefulness did not significantly influence user satisfaction (H1: *β* = 0.06, *p* > 0.05), diverging from earlier ISCM-based research (Bhattacherjee, 2001). This deviation may be attributed to vocational learners prioritizing experiential and emotional aspects—such as enjoyment and interpersonal support—over purely utilitarian assessments. In platforms where use is obligatory, functional adequacy may be viewed as a minimum requirement that is insufficient to generate satisfaction on its own. Similarly, perceived ease of use did not exert a direct influence on continuance intention (H7: *β* = 0.08, *p* > 0.05). While ease of use does contribute indirectly via perceived usefulness (as shown in H6), its standalone effect appears diminished, possibly due to increased digital literacy and baseline usability expectations among students. Particularly in vocational settings, where platform familiarity is often developed through repeated exposure, usability may no longer serve as a differentiator but instead be taken for granted.

Perceived trust also failed to predict continuance intention significantly (H10: *β* = 0.04, *p* > 0.05). While trust contributed to perceived usefulness (H8), its lack of direct influence suggests that in institutionally controlled environments, students may not equate trust with volitional platform use. Instead, a trust may operate as a background condition—necessary for credibility—but insufficient to motivate sustained behavioral engagement in mandatory systems. Finally, system quality did not show a significant effect on user satisfaction (H13: *β* = 0.05, *p* > 0.05), which contrasts with assertions from the Information Systems Success Model (DeLone and McLean, 1992). A plausible interpretation is that vocational students assume a baseline level of technical performance, shifting their evaluative focus toward pedagogical responsiveness, content relevance, emotional support, and personalization. In such contexts, system quality may function more as a hygiene factor than a driver of satisfaction.

#### 5.2.3 Contradictory hypotheses (negative relationships)

A theoretically unexpected yet statistically significant finding emerged regarding the relationship between perceived trust and user satisfaction (H9: *β* = −0.13, *p* < 0.05). Contrary to the widely accepted positive association in existing literature (e.g., Chen et al., 2021; He et al., 2023; Yang et al., 2024), this study observed a negative path coefficient. Interestingly, this outcome aligns with findings from Ojeme et al. (2019), who reported that in constrained institutional settings, elevated trust levels may paradoxically decrease satisfaction.

A key explanation centers on the expectation-disconfirmation mechanism. In contexts where platform use is mandatory—such as vocational colleges—students often associate institutional platforms with credibility and authority, leading to heightened expectations for content quality, interactivity, and usability. When these expectations are unmet, cognitive dissonance arises, resulting in dissatisfaction despite initial trust (Ficapal-Cusí et al., 2024). This phenomenon is further corroborated by Zhang and Chen (2021), who found that vocational learners expressed frustration with institutional platforms due to issues such as content overload, excessive task demands, and insufficient interactivity—factors that diminish perceived value even when institutional trust is present. This paradoxical relationship may also reflect broader organizational dynamics. Gong et al. (2024) demonstrated that procedural justice in system design fosters psychological ownership, which in turn mitigates dissatisfaction even in mandatory-use contexts. Moreover, as Chi (2019) noted, trust and usability are necessary but insufficient conditions for satisfaction when learner autonomy and psychological support are overlooked. In vocational education settings where platform deployment often follows top-down mandates, the absence of user-centered design can erode the positive effects typically associated with trust.

Collectively, these findings highlight the conditional nature of trust in technology adoption models. In compulsory-use environments, trust may inflate user expectations without guaranteeing enhanced satisfaction. Therefore, managing expectations through transparent communication, participatory goal setting, and empathetic platform design becomes essential for aligning institutional trust with actual user experiences and promoting positive engagement outcomes.

### 5.3 Practical implications

The empirical findings offer actionable insights for platform developers and educational administrators who aim to enhance learner engagement in digital environments, particularly within vocational education contexts.

First, the strong predictive effects of user satisfaction and perceived enjoyment suggest that platform design should prioritize affective and motivational features. Integrating gamified elements (Wang et al., 2022; Kashive and Mohite, 2023), interactive multimedia content (Alshammari and Alshammari, 2024; Tarigan et al., 2023), and self-paced, adaptive learning modules (Mutawa et al., 2023; Tan, 2019) can significantly boost emotional engagement and intrinsic motivation—two critical drivers of continued platform use in non-voluntary settings. Second, educational institutions must implement robust support systems that cater not only to academic progress but also to learners’ emotional wellbeing. Mechanisms such as real-time feedback, responsive technical support, and adaptive scaffolding should be complemented by social features like peer collaboration, group discussions, and community-building projects. These initiatives foster a sense of connection and belonging, which has been positively associated with sustained platform engagement (Saura et al., 2020; Alshammari and Alshammari, 2024). Finally, consistent with Self-Determination Theory and recent findings by Ficapal-Cusí et al. (2024), ensuring emotional autonomy is vital. Platforms should empower students to regulate their own learning pace, customize their pathways, and receive personalized encouragement and feedback. Such features not only enhance user satisfaction but also promote long-term adoption by aligning digital experiences with individual psychological needs.

In sum, these recommendations emphasize the importance of designing emotionally responsive and student-centered digital ecosystems, especially in vocational settings where technology use is mandatory. Balancing cognitive efficiency with emotional resonance is key to fostering meaningful and sustained engagement with innovative learning platforms.

## 6 Conclusion

This study offers valuable theoretical and empirical insights into the determinants of students’ continued engagement with online learning platforms in vocational education settings. By integrating cognitive and affective variables within an extended framework combining the Technology Acceptance Model (TAM) and the Information Systems Continuance Model (ISCM), the findings highlight the pivotal roles of perceived enjoyment, user satisfaction, and perceived usefulness in fostering sustained platform use. These results align with previous research (e.g., Mora-Cruz et al., 2023), which emphasized the importance of emotional engagement and satisfaction in predicting successful digital learning outcomes.

Among the significant findings, perceived enjoyment emerged as the strongest predictor of continuance intention, reaffirming the relevance of Self-Determination Theory (SDT) and underscoring the influence of intrinsic motivation on behavioral persistence. This highlights the need to account for emotional engagement when designing learning technologies for vocational learners, who may rely more heavily on affective drivers in mandatory-use contexts. Unexpectedly, the study revealed a negative relationship between perceived trust and user satisfaction—challenging widely held assumptions in TAM-based research. This finding is supported by prior literature (e.g., Ojeme et al., 2019; Ficapal-Cusí et al., 2024), which suggests that elevated institutional trust can lead to heightened expectations. When platform performance fails to meet these expectations, students may experience dissonance and dissatisfaction. Evidence from the Chinese vocational education sector further supports this interpretation, with students expressing frustration over content overload and low interactivity despite trusting institutional platforms (Zhang and Chen, 2021; Chi, 2019).

While the study contributes to refining technology acceptance theory in underexplored educational contexts, certain limitations should be acknowledged. The sample was limited to Chinese vocational college students, which may affect the generalizability of the findings. Moreover, the use of self-reported, cross-sectional data restricts the ability to infer causality. Future research should consider longitudinal or experimental designs, explore habit formation mechanisms, and extend the model to diverse educational settings. In sum, this study enhances our understanding of technology adoption in vocational education by integrating emotional and cognitive dimensions into extended TAM frameworks. Practically, the findings suggest that improving interactivity, enabling personalized learning pathways, and proactively managing students’ expectations are critical strategies for promoting trust, satisfaction, and long-term engagement in mandatory digital learning environments.

## Data Availability

The raw data supporting the conclusions of this article will be made available by the authors, without undue reservation.
